# Ozone at low concentration modulates microglial activity in vitro: A multimodal microscopy and biomolecular study

**DOI:** 10.1002/jemt.24233

**Published:** 2022-09-21

**Authors:** Maria Assunta Lacavalla, Chiara Rita Inguscio, Barbara Cisterna, Paolo Bernardi, Manuela Costanzo, Mirco Galiè, Ilaria Scambi, Osvaldo Angelini, Gabriele Tabaracci, Manuela Malatesta

**Affiliations:** ^1^ Department of Neurosciences, Biomedicine and Movement Sciences, Anatomy and Histology Section University of Verona Verona Italy; ^2^ San Rocco Clinic Montichiari Italy

**Keywords:** fluorescence microscopy, nuclear factor erythroid 2‐related factor 2, oxygen‐ozone therapy, scanning electron microscopy, transmission electron microscopy

## Abstract

Oxygen‐ozone (O_2_‐O_3_) therapy is an adjuvant/complementary treatment based on the activation of antioxidant and cytoprotective pathways driven by the nuclear factor erythroid 2‐related factor 2 (Nrf2). Many drugs, including dimethyl fumarate (DMF), that are used to reduce inflammation in oxidative‐stress‐related neurodegenerative diseases, act through the Nrf2‐pathway. The scope of the present investigation was to get a deeper insight into the mechanisms responsible for the beneficial result of O_2_‐O_3_ treatment in some neurodegenerative diseases. To do this, we used an integrated approach of multimodal microscopy (bright‐field and fluorescence microscopy, transmission and scanning electron microscopy) and biomolecular techniques to investigate the effects of the low O_3_ concentrations currently used in clinical practice in lipopolysaccharide (LPS)‐activated microglial cells human microglial clone 3 (HMC3) and in DMF‐treated LPS‐activated (LPS + DMF) HMC3 cells. The results at light and electron microscopy showed that LPS‐activation induced morphological modifications of HMC3 cells from elongated/branched to larger roundish shape, cytoplasmic accumulation of lipid droplets, decreased electron density of the cytoplasm and mitochondria, decreased amount of Nrf2 and increased migration rate, while biomolecular data demonstrated that Heme oxygenase 1 gene expression and the secretion of the pro‐inflammatory cytokines, Interleukin‐6, and tumor necrosis factor‐*α* augmented. O_3_ treatment did not affect cell viability, proliferation, and morphological features of both LPS‐activated and LPS + DMF cells, whereas the cell motility and the secretion of pro‐inflammatory cytokines were significantly decreased. This evidence suggests that modulation of microglia activity may contribute to the beneficial effects of the O_2_‐O_3_ therapy in patients with neurodegenerative disorders characterized by chronic inflammation.

**Highlights:**

- Low‐dose ozone (O_3_) does not damage activated microglial cells in vitro
- Low‐dose O_3_ decreases cell motility and pro‐inflammatory cytokine secretion in activated microglial cells in vitro
- Low‐dose O_3_ potentiates the effect of an anti‐inflammatory drug on activated microglial cells

## Graphical abstract

Low‐dose ozone (O_3_) does not damage activated microglial cells in vitro, but is able to decrease cell motility and pro‐inflammatory cytokine secretion, and potentiate the effect of anti‐inflammatory drugs. Modulation of microglia activity may contribute to the beneficial effects of O_3_ therapy in patients with neurodegenerative disorders characterized by chronic inflammation.

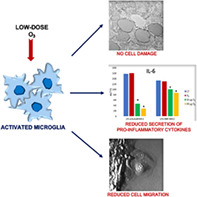

## 1 INTRODUCTION

Oxygen‐ozone (O_2_‐O_3_) therapy is a modestly invasive procedure used in medicine as an adjuvant/complementary treatment for a variety of diseases (Bocci, 2012; Delgado‐Roche et al., 2017; Elvis & Ekta, 2011; Re et al., 2008). O_3_ is a highly unstable gas that quickly dissolves and decomposes in the body fluids (being tenfold more water‐soluble than O_2_). O_3_ therefore acts as a pro‐drug because it does not react directly on the cells but gives rise to molecular messengers that, in turn, diffuse in the whole organism (some of them being even able to pass the blood brain barrier (Masan et al., 2021)) thus activating multiple pathways responsible for the therapeutic response (Sagai & Bocci, 2011). Molecular evidence shed light on some basic biological mechanisms responsible for the dose‐dependent effects of O_3_ exposure (Sagai & Bocci, 2011; Viebahn‐Haensler & Fernández, 2021): high O_3_ concentrations induce an inflammatory response by activating the redox‐sensitive nuclear factor kappa‐light‐chain‐enhancer of activated B cells, which promotes the transcription of pro‐inflammatory cytokines and, in turn, the expression of several proteins involved in the antioxidant response (Sagai & Bocci, 2011); on the contrary, low O_3_ concentrations induce a moderate oxidative stress that stimulates the transcription of Antioxidant Response Elements‐driven genes through the translocation of the nuclear factor erythroid 2‐related factor 2 (Nrf2) from the cytoplasm to the cell nucleus, where it promotes the transcription of several genes involved in the antioxidant response (Galiè et al., 2018). Therefore, the therapeutic efficacy of low‐dose O_3_ would rely on the induction of an oxidative “eustress” (Niki, 2016) that stimulates the antioxidant cell defense pathways via Nrf2 activation (Galiè et al., 2019) without inducing injury or inflammation.

Interestingly, there is increasing evidence on the role of the Nrf2 pathway in reducing oxidative stress and inflammation in neurodegenerative conditions, thus making the Nrf2 a promising therapeutic target for these diseases (Buendia et al., 2016; Dinkova‐Kostova et al., 2018; Johnson & Johnson, 2015; Liu et al., 2021; Lu et al., 2016; McBean et al., 2017; Miller et al., 2019; Robledinos‐Antón et al., 2019; Shaw & Chattopadhyay, 2020). Consistently, some drugs, such as dimethyl fumarate (DMF) (Scannevin et al., 2012), are able to reduce inflammation in neurodegenerative diseases acting through the Nrf2 pathway.

In the present investigation, we focused our attention on microglial cells in the frame of a basic research aimed at unveiling the biological mechanisms accounting for the therapeutic efficacy of low‐dose O_3_ on different cell types. Microglia are resident phagocytes and innate immune cells in the central nervous system (CNS), where they contribute to the homeostasis and rapidly activate in response to noxious stimuli, thus playing a primary role in inflammatory processes (Prinz et al., 2019; Wolf et al., 2017; Woodburn et al., 2021).

To ensure controlled and standardized experimental conditions, we selected as an in vitro system the human microglial clone 3 (HMC3) cell line, which is widely employed for basic studies (Dello Russo et al., 2018). HMC3 cells were administered the low O_3_ concentrations currently used in clinical practice after activation with lipopolysaccharide (LPS) (which induces neuroinflammatory responses and upregulates the expression of pro‐inflammatory cytokines (Lu et al., 2021)); to mimic the effect of O_3_ on activated microglia under a pharmacological anti‐inflammatory treatment, we also treated HMC3 cells with the same gas concentrations after both LPS activation and DMF administration (Scannevin et al., 2012). To evaluate the effects of O_3_ on the structural and functional features of HMC3 cells we used an integrated approach of multimodal microscopy (bright‐field and fluorescence microscopy, transmission and scanning electron microscopy) and biomolecular techniques.

## 2 MATERIALS AND METHODS

### 2.1 Cell culture and treatment

Human microglial clone 3 cell line, HMC3 (ATCC), were chosen for the present study as a suitable in vitro system widely used in investigations on neurodegenerative diseases (Dello Russo et al., 2018). HMC3 cells were grown in Minimum Essential Medium supplemented with 11% (vol/vol) fetal bovine serum, 1% (wt/vol) glutamine, 100 U of penicillin and 100 g/ml streptomycin (all reagents were purchased from Gibco, Walthem, MA, USA) at 37°C in a 5% CO_2_ humidified atmosphere. At sub‐confluence, the cells were trypsinized with 0.25% trypsin in phosphate buffered saline (PBS) containing 0.05% EDTA (Gibco), and seeded for specific analyses.

Cells were exposed to O_2_–O_3_ gas mixtures produced from medical‐grade O_2_ by an OZO2 FUTURA apparatus (Alnitec, Cremosano, CR, Italy), which allows photometric real‐time control of gas flow rate and O_3_ concentration. O_3_ was used at the concentrations of 10 and 20 μg O_3_/ml O_2_ because they are currently administered in the clinical practice. In addition, these concentrations proved be non‐toxic for various cultured cells and tissues (Cappellozza et al., 2021; Cisterna et al., 2020; Cisterna et al., 2021; Costanzo et al., 2018; Costanzo et al., 2015; Scassellati et al., 2017). Concentrations of 30 and 50 μg O_3_/ml O_2_ were used as highly oxidizing conditions.

Pure O_2_ was used to distinguish the effect of O_3_ from O_2_ in the context of the O_2_–O_3_ mixtures. Cells undergoing the same handling as gas‐treated cells but without exposure to O_2_ or O_2_–O_3_ gas were considered as control (CT).

As for cells grown adhering to glass slides, two coverslips were placed in a 50 ml polypropylene syringe with 16 ml culture medium, then 16 ml of gas was added into the syringe using a sterile filter (Alnitec, Cremosano, CR, Italy), and the medium was gently mixed with the gas for 10 min (Costanzo et al., 2015). As for cells treated in suspension, samples of 4 × 10^6^ cells were suspended in 10 ml medium into a 20 ml syringe, then 10 ml of gas was added into the syringe and gently mixed with the gas for 10 min (Larini et al., 2003).

For mitotic index and S‐phase assessment, wound healing assay, TEM analyses, and SEM analyses, the cells were seeded on glass slides in multi‐well microplates, let to adhere for at least 24 h and then submitted to gas treatment. For cytotoxicity evaluation, RT‐qPCR and cytokine assays, cells were treated in suspension. Then, for methyl thiazolyl tetrazolium (MTT) assay, cells were seeded in 96‐multi‐well plate after gas treatment, and analysed. For cytokine evaluation, cells were seeded after gas treatment in 24‐multi‐well plates and, after 24 h, the medium was collected and stored at −80°C.

Before gas treatment, some HMC3 samples were incubated with 1 μg/ml LPS for 24 h as previously reported (Dello Russo et al., 2018) to induce cell activation. Some other HMC3 samples were incubated with both 4 μM DMF and 1 μg/ml LPS for 24 h in order to counteract cell activation with an antioxidant drug (Scannevin et al., 2012). Sample of HMC3 cells non‐activated was used as reference condition to verify the efficacy of LPS activation and DMF treatment.

### 2.2 Cell viability assay

The effect of gas treatment was evaluated by the MTT assay. Cells were seeded in flat‐bottom 96 multiwell plates at the density of 5 × 10^3^ cells/well. Five wells for each condition were seeded.

MTT assay was performed at 24, 48, and 72 h after gas treatment in LPS‐activated and LPS + DMF HMC3 cells. To evaluate the effect of higher O_3_ concentrations on cell viability, samples of non‐activated cells were exposed to 30 and 50 μg O_3_/ml O_2_. Briefly, the medium was replaced with 100 μl of 0.5 mg/ml MTT (Sigma, Italy) in culture medium and incubated for 4 h at 37°C in a cell culture incubator. Then, MTT solution was removed, formazan crystals were dissolved in 100 μl of dimethyl sulfoxide (DMSO) and the absorbance was measured at 570 nm. The percentage of cell viability was calculated.

Cell death for the highly oxidizing conditions of 30 and 50 μg O_3_/ml O_2_ was estimated at the same times of MTT assay (24, 48, and 72 h after gas treatment) staining the cells with 0.1% Trypan blue for 2 min. The cells were observed using a Leica DM IL inverted microscope equipped with 20× objective lens.

### 2.3 Mitotic index

The percentage of mitotic cells was assessed 48 h after treatment in LPS‐activated and LPS + DMF HMC3 cells, as a measure of the cell proliferation rate. Non‐activated cell sample was also considered as basal condition. The cells (2 × 10^4^ seeded cells per 24 mm × 24 mm slides) were fixed with 70% ethanol for 30 min, washed with PBS and stained for deoxyribonucleic acid (DNA) with 0.1 μg/ml Hoechst 33342 (Abcam, Cambridge, United Kingdom) in PBS for 10 min. The samples were finally mounted in PBS/glycerol (1:1).

For observation, an Olympus BX51 microscope (Olympus Italia S.r.l., Segrate, MI, Italy) equipped with a 100 W mercury lamp was used under the appropriate light excitation and emission conditions for Hoechst 33342. Images were recorded with a QICAM Fast 1394 Digital Camera (QImaging, Surrey, BC, Canada) and processed with Image‐Pro Plus software (Media Cybernetics, Inc., Rockville, MD, USA).

### 2.4 S‐phase evaluation

In order to assess cell proliferation rate, HMC3 S‐phase evaluation was performed in LPS‐activated and LPS + DMF cells 48 h after treatment. Non‐activated cell sample was also considered as basal condition. After 2 × 10^4^ cells were seeded on 24 mm × 24 mm slides, pulse‐labeled with 20 μM Bromodeoxyuridine (BrdU) (Sigma‐Aldrich, St. Louis, MO, USA) at 37°C for 30 min and fixed with 70% ethanol. To partially denature DNA, cells were incubated with 2 N HCl for 20 min at room temperature, then neutralized for 3 min with 0.1 M sodium tetraborate (pH 8.2) (Sigma‐Aldrich). Samples were washed with PBS and permeabilized with PBS containing 0.1% bovine serum albumin and 0.05% Tween‐20 (Sigma‐Aldrich) for 15 min, then incubated with a mouse monoclonal antibody recognizing BrdU (BD Diagnostics, Franklin Lakes, NJ, USA) diluted 1:20 in PBS for 1 h. Following two washes with PBS, cells were incubated with Alexa Fluor 488‐conjugated anti‐mouse secondary antibody (Molecular Probes, Invitrogen, Milan, MI, Italy) diluted 1:200 for 1 h, washed with PBS twice and DNA stained for 10 min with 0.1 μg/ml Hoechst 33342 (Abcam, Cambridge, United Kingdom) in PBS. Samples were finally mounted with PBS/glycerol 1:1 solution.

BrdU‐positive cells percentage was assessed in 30 randomly selected fields (40× magnification) for every experimental condition. Observation of samples was performed using an Olympus BX51 microscope (Olympus Italia S.r.l., Segrate, MI, Italy) equipped with a 100 W mercury lamp, under the following conditions: 450–480 nm excitation filter (excf), 500 nm dichroic mirror (dm), and 515 nm barrier filter (bf) for Alexa Fluor 488; 330–385 nm excf, 400 nm dm, and 420 nm bf, for Hoechst 33342. Images were acquired with a QICAM Fast 1394 Digital Camera (QImaging, Surrey, BC, Canada) and processed with Image‐Pro Plus software (Media Cybernetics, Inc., Rockville, MD, USA).

### 2.5 Wound healing assay

For the wound healing assay, 2 × 10^5^ cells per well were seeded on 24 mm × 24 mm slides. After 24 h, the confluent cell monolayers were scratched with a sterile pipette tip and then treated with gas. To evaluate cell migration, images at 4× magnification were taken at 0, 6, 24, and 48 h post‐treatment using an inverted microscope (Leica DMIL, Leica Microsystems S.r.l., Buccinasco, MI, Italy) equipped with a camera (Optika Microscopes, Ponteranica, BG, Italy). The scratched area free of cells was measured in four randomly chosen fields in three independent experiments, for a total of 12 fields per sample. The value of the cell‐free area was expressed as a percentage of the value at time 0 (considered as 100%).

### 2.6 Scanning electron microscopy (SEM)

For SEM examination, 2 × 10^4^ cells were seeded on round slides of 20 mm in diameter. After 24 h, the cell monolayers of LPS‐activated and LPS + DMF samples were scratched as described above and treated with gas. Non‐activated cell sample was also considered as basal condition. After 24 h post‐treatment, the cells were fixed with 2.5% glutaraldehyde in PBS at 4°C for 2 h, post‐fixed with 1% OsO_4_ at 4°C for 1 h, and dehydrated with ethanol. Then, the cell monolayers were dehydrated with a critical point dryer (CPD 030, BAL‐TEC AG, Balzers, Liechtenstein), mounted on metallic specimen stubs and sputter‐coated with gold (MED 010, BAL‐TEC AG). SEM observations were performed by an XL30 ESEM (FEI Italia S.r.l., Milan, Italy). Using ImageJ software (NIH), the surface length of 15 cells per sample facing the scratch was measured. Measure was made by including and excluding cell protrusions and the ratio between the two values gave the index of cell surface irregularity (the higher the value, the rougher the cell).

### 2.7 Transmission electron microscopy (TEM)

Morphological and immunocytochemical analyses were carried out at TEM in order to analyze the effects of the exposure to low O_3_ concentrations on the fine cell features and Nrf2 nuclear translocation. Based on our previous investigations (Galiè et al., 2018), the effects were evaluated 24 h after gas treatment, in order to clearly detect morphological changes and Nrf2 translocation on the transcriptional sites. Non‐activated cell sample was also considered as basal condition. The cells (2 × 10^4^ cells per well) were seeded on round slides of 20 mm in diameter. After 24 h, the cell monolayers were treated with gas. After 24 h post‐treatment, the cells were fixed with 2.5% glutaraldehyde and 2% paraformaldehyde in 0.1 M phosphate buffer, pH 7.4, at 4°C for 1 h, washed, post‐fixed with 1% OsO_4_ at 4°C for 30 min, dehydrated with acetone and embedded in Epon as monolayer (Costanzo & Malatesta, 2019).

For ultrastructural morphology, ultrathin sections were collected and stained with Reynolds lead citrate. For immunocytochemistry, ultrathin sections were collected and immunolabeled. Briefly, sections were floated on normal goat serum diluted 1:100 in PBS, incubated overnight at 4°C with the anti‐Nrf2 antibody (Abcam #ab62352, Cambridge, United Kingdom) diluted 1:2 with PBS containing 0.1% bovine serum albumin (Fluka, Buchs, Switzerland) and 0.05% Tween 20. Sections were then floated on normal goat serum and incubated for 30 min with a goat anti‐rabbit IgG secondary antibody conjugated with 12‐nm gold particles (Jackson ImmunoResearch Laboratories Inc., West Grove, PA, USA), diluted 1:20 in PBS. After rinsing with PBS and water, the sections were finally air‐dried and weakly stained with Reynolds lead citrate for 1 min. As immunostaining controls, the primary antibody was omitted.

The samples were observed in a Philips Morgagni transmission electron microscope (FEI Company Italia Srl, Milan, Italy) operating at 80 kV; a Megaview III camera (FEI Company Italia Srl) was used for image acquisition.

Quantitation of anti‐Nrf2 immunolabeling was performed by estimating the gold particle density on sections treated in the same run: the area of nucleoplasmic regions and resin regions (as an intra‐sample negative control) was measured on 15 micrographs (28,000×) per sample. Background evaluation was performed in sections processed for immunocytochemistry without the primary antibody. In each measured area, the gold particles were counted manually and the labeling density (i.e., the number of gold particles/μm^2^ of nucleoplasm) was calculated.

### 2.8 Real‐time quantitative polymerase chain reaction

Ribonucleic acid (RNA) was extracted from LPS‐activated and LPS + DMF HMC3 samples 24 h after the gas exposure by using the Qiagen RNeasy Plus mini kit (ref. 74134) (QiagenS.r.l., Milan, Italy). cDNA was generated by SuperScript™ III Reverse Transcriptase (Invitrogen, cat. no. 18080093) (Thermo Fisher Scientific Inc., Waltham, MA, USA) and amplified at qPCR with Applied Biosystems™ SYBR™ Green PCR Master Mix (Applied Biosystems™ 4309155) (Thermo Fisher Scientific Inc.) using two distinct sets of primers specific for human Heme oxygenase 1 (Hmox1) (primers set 1: Forw: CCTAAACTTCAGAGGGGGCG, Rev: GACAGCTGCCACATTAGGGT; primers set 2: Forw: AGTCTTCGCCCCTGTCTACT, Rev: CTTCACATAGCGCTGCATGG). The Applied Biosystems Step‐One Real‐Time PCR System was used for amplification (Thermo Fisher Scientific Inc.).

### 2.9 IL‐6, TNF‐*α*, and IL‐13 secretion

The amount of IL‐6, TNF‐*α*, and Interleukin‐13 (IL‐13) secreted was evaluated in the culture medium of LPS‐activated and LPS + DMF HMC3 cells, 24 h after the gas treatment (Dello Russo et al., 2018). Non‐activated cell sample was also considered as basal condition. The pro‐inflammatory IL‐6 and TNF‐*α* are known to be produced by HMC3 (Dello Russo et al., 2018). Recently it has been demonstrated that HMC3 cells are able to synthesize and secrete also small amounts of the anti‐inflammatory IL‐13 (Caruso et al., 2021; Pallio et al., 2021).

For each sample, 2 × 10^4^ cells per 24‐multi‐well plate were seeded after gas treatment; experiments were performed in duplicate. The medium collected from each cell sample was centrifuged at 1500 g for 15 min, and stored at −80°C. Quantitation of IL‐6, TNF‐*α*, and IL‐13 were quantified using a Luminex Bio‐Rad Bio‐Plex 100 (Bio‐Rad Laboratories, Segrate, MI, Italy) and the Bio‐Plex Manager software, v6.0. A total of 50 μl aliquots of undiluted medium were placed in a 96‐well plate, beads conjugated with fluorophores and antibodies against IL‐6, TNF‐*α*, and IL‐13 were added and, after appropriate incubation and washing, the plate was read by the Luminex system. Samples were run in duplicate.

### 2.10 Statistical analysis

For each variable mean value ± SD were calculated. For statistical analysis of MTT assay, wound healing assay, and cytokine secretion, the Kruskal–Wallis non‐parametric test followed by the Mann–Whitney test for pairwise comparison was applied. For statistical comparison of the mitotic index and the index of cell surface irregularity, the one‐way analysis of variance (ANOVA) test followed by Bonferroni's post‐hoc test was used. A *p* value ≤0.05 indicated statistical significance.

## 3 RESULTS

### 3.1 Cell viability assay

**FIGURE 1:**
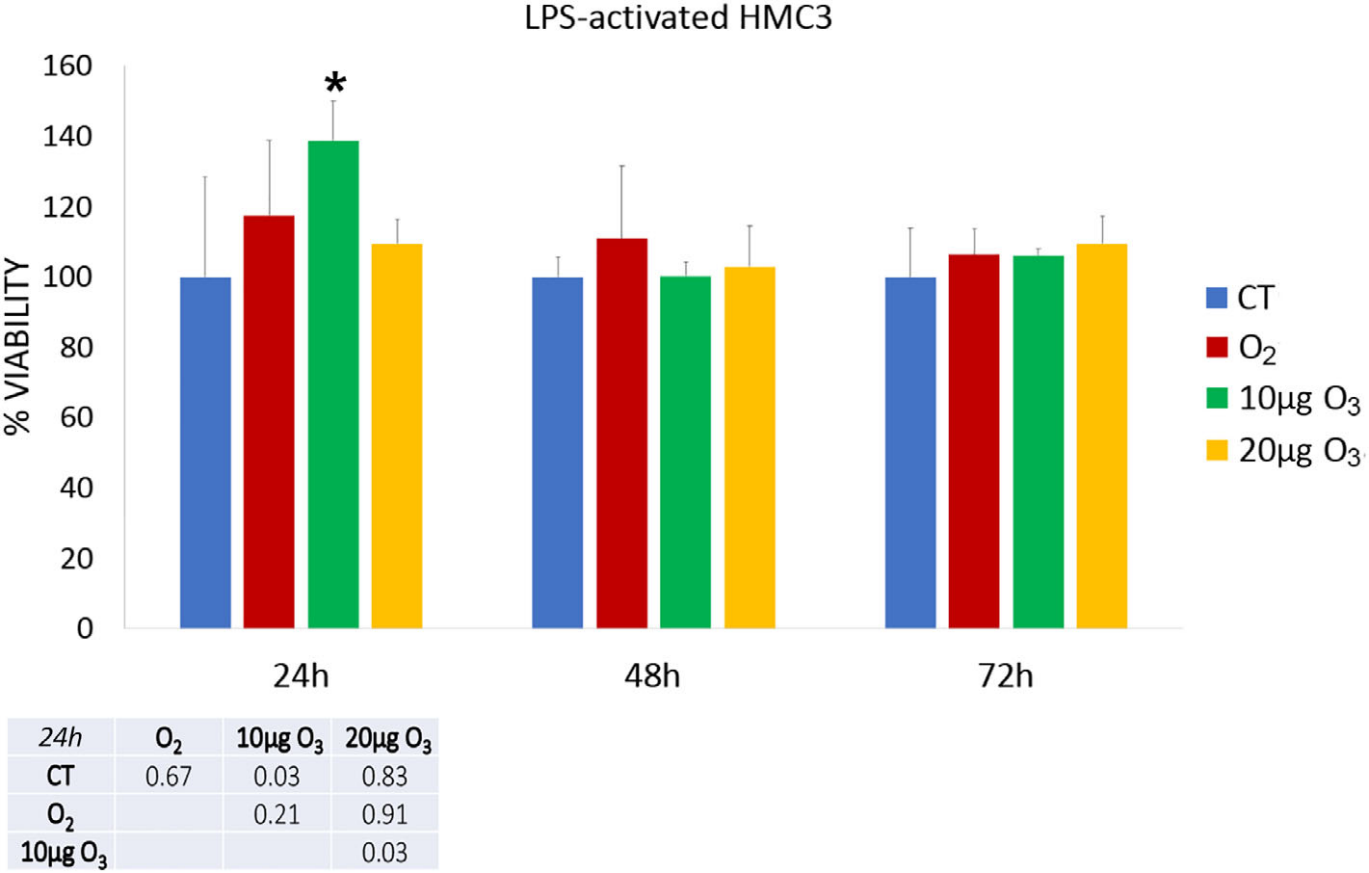
Cell viability 24, 48, and 72 h after O_2_‐O_3_ treatment in LPS‐activated HMC3 cells as assessed by the MTT assay. Histograms show the mean values ± SD of percentage of cell viability; the table reports the *p* values for all the comparisons made. Asterisk (*) indicates the statistically significant difference from the corresponding CT sample (*p* < 0.05). HMC3, human microglial clone 3; LPS, lipopolysaccharide

**FIGURE 2:**
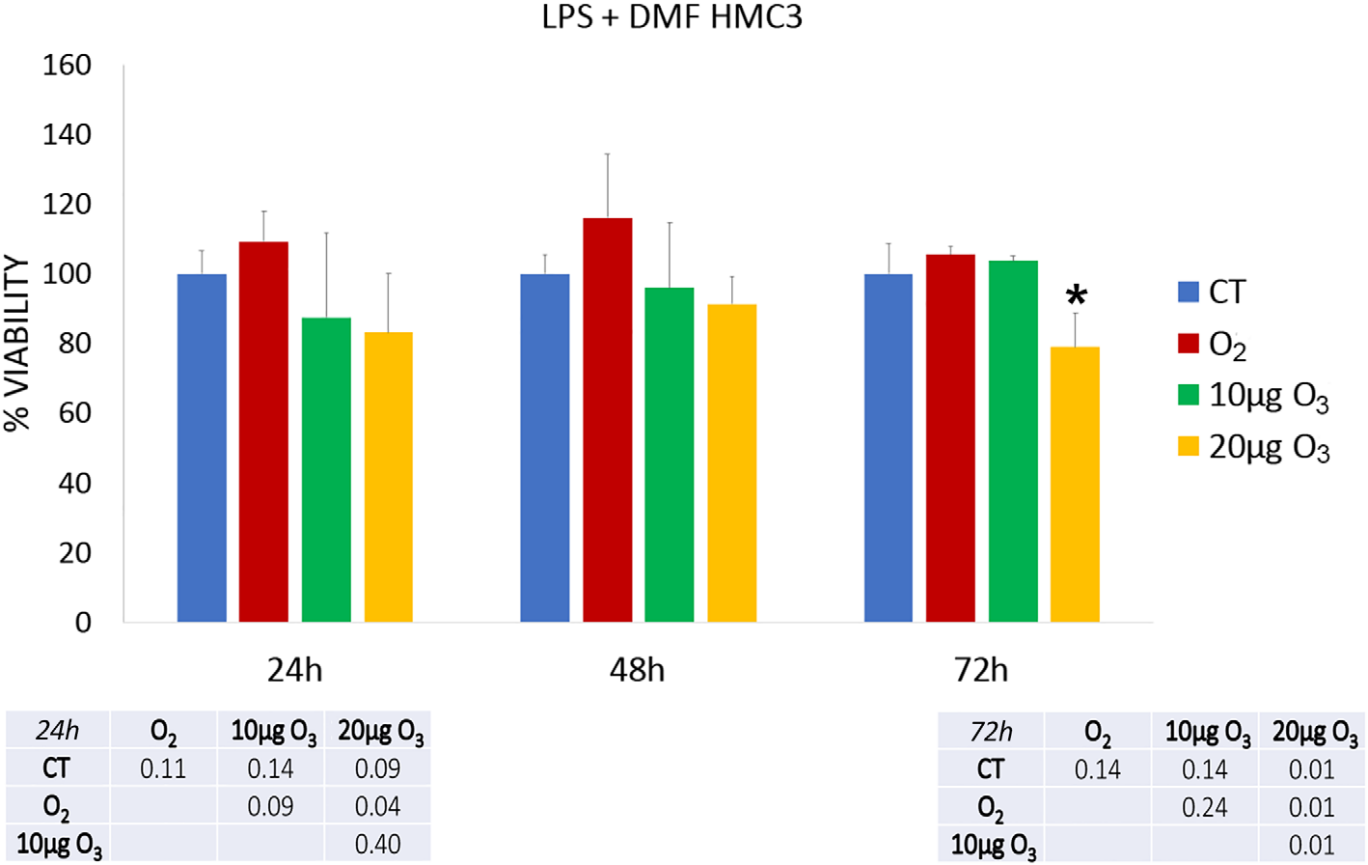
Cell viability 24, 48, and 72 h after the treatment in LPS + DMF HMC3 cells as assessed by the MTT assay. Histograms show the mean values ± SD of percentage of cell viability; the table reports the *p* values for all the comparisons made. Asterisk (*) indicates the statistically significant difference from the respective CT sample (*p* < 0.05). DMF, dimethyl fumarate; HMC3, human microglial clone 3; LPS, lipopolysaccharide

The effect of gas treatment on cell viability was assessed in LPS‐activated (Figure 1) and LPS + DMF HMC3 cells (Figure 2).

After 24 h of gas exposure, 10 μg O_3_ LPS‐activated HMC3 samples showed a statistically significant increase in cell viability in comparison with CT and 20 μg O_3_‐treated cells. After both 48 and 72 h, no statistically significant difference resulted among the LPS‐activated samples (*p* = 0.68 and *p* = 0.75, respectively).

In LPS + DMF cells, gas exposure did not change significantly cell viability in comparison with CT samples (a statistically significant decrease was only observed in 20 μg O_3_ LPS + DMF cells in comparison with O_2_‐treated cells). After 48 h of post‐treatment, no statistically significant difference was found among the LPS + DMF samples (*p* = 0.17). At 72 h, 20 μg O_3_ LPS + DMF cells showed significantly lower cell viability in comparison to all the other samples.

The exposure to 30 and 50 μg O_3_/ml O_2_ induced a drastic decrease of the cell viability already 24 h post‐treatment as verified by the trypan blue test (not shown). The concentrations of 30 and 50 μg O_3_/ml O_2_ were indeed excluded from the experimentation.

### 3.2 Cell proliferation

**FIGURE 3:**
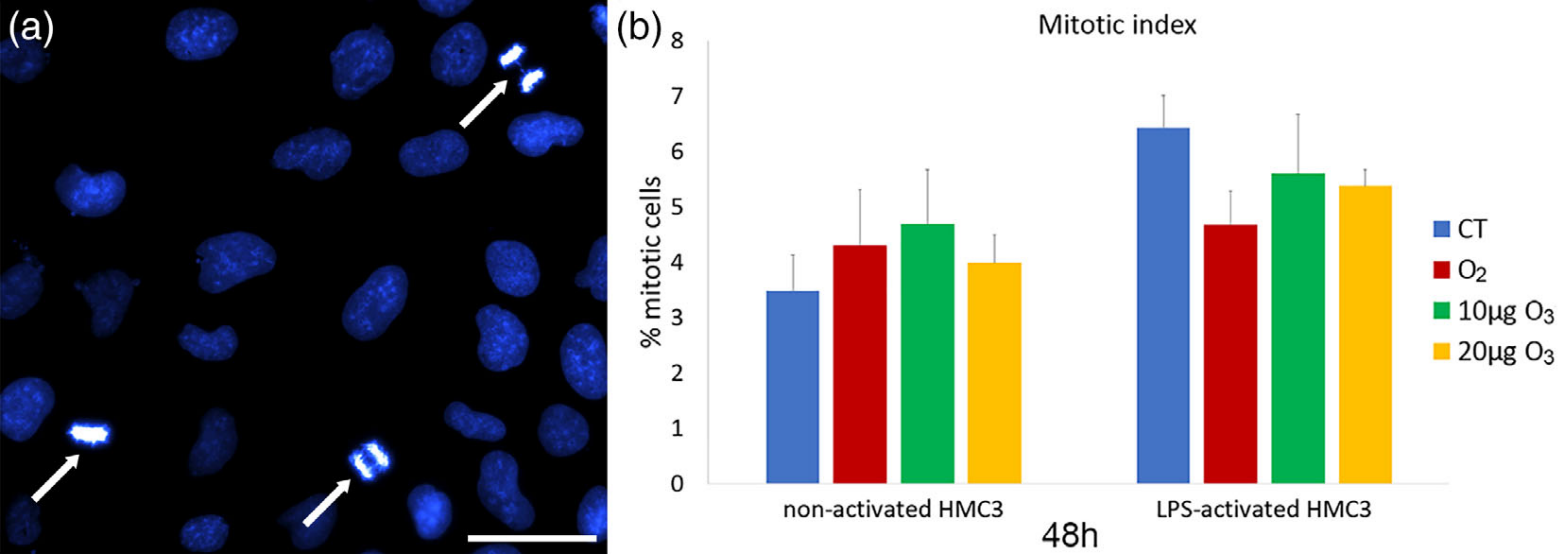
(a) Representative image at fluorescence microscopy of HMC3 cells stained for DNA with Hoechst 33342. Note the mitotic cells (arrows). Bar, 100 μm. (b) Mean values ± SD of percentage of mitotic cells at 48 h after the treatment in LPS‐activated and LPS + DMF HMC3 cells. DMF, dimethyl fumarate; HMC3, human microglial clone 3; LPS, lipopolysaccharide

The proliferation activity of HMC3 cells was assessed by evaluating the mitotic index and the percentage of S‐phase positive cells by BrdU incorporation. Mitotic index was assessed in non‐activated HMC3 cells (3.49 ± 0.64, as basal condition) and compared with CT samples of LPS‐activated and LPS + DMF cells, revealing no statistically significant difference (*p* = 0.51). The percentage of mitotic cells observed 48 h after treatment in LPS‐activated and LPS + DMF cells showed no statistically significant difference between CT and treated cells (*p* = 0.80, *p* = 0.78, and *p* = 0.98, respectively) (Figure 3).

**FIGURE 4:**
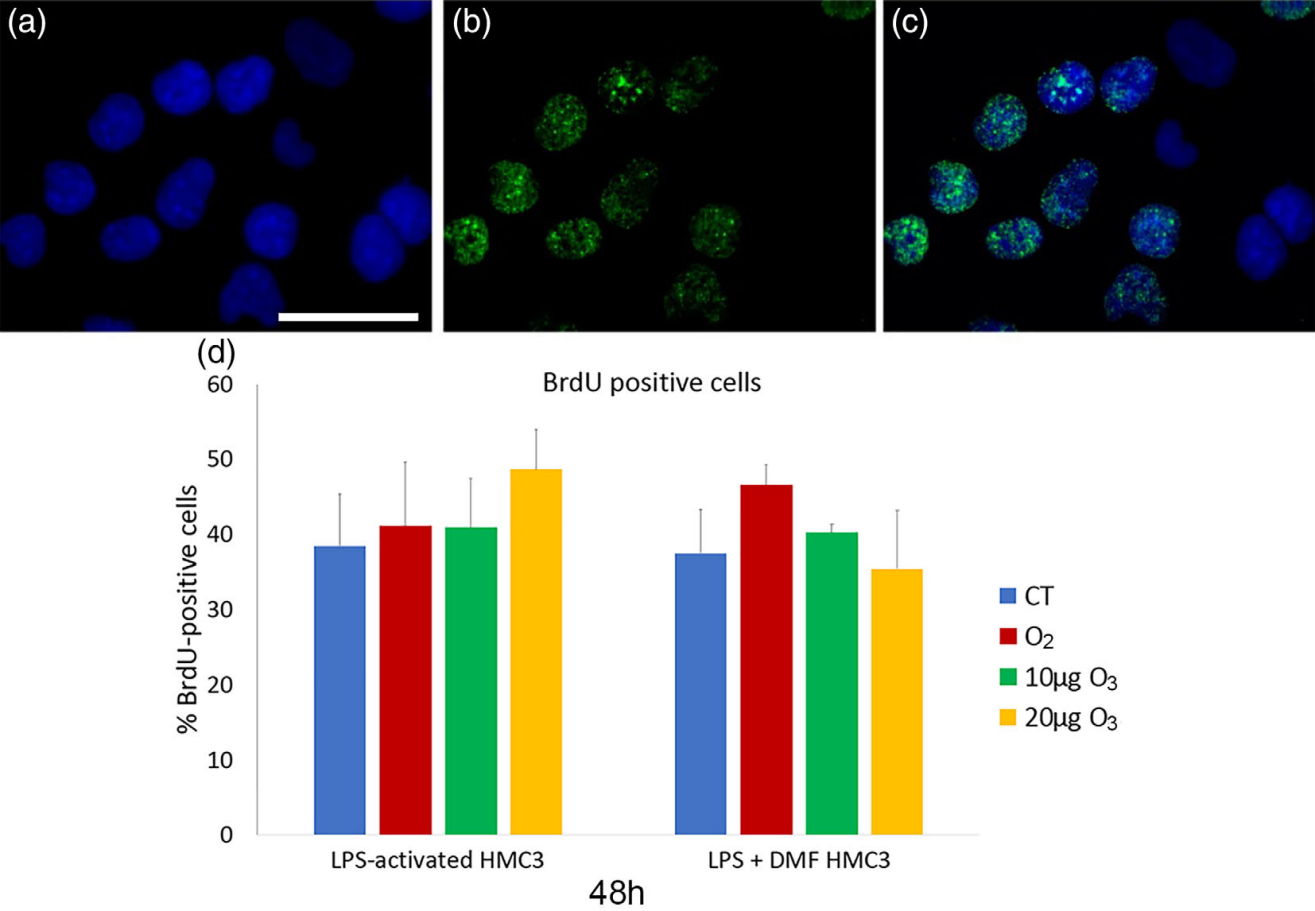
Representative fluorescence microscopy images of HMC3 cells stained for DNA with Hoechst 33342 (blue) (a), immunolabeled for BrdU (green) (b), and merged (c). Bar, 100 μm. (d) Mean values ± SD of percentages of BrdU‐positive cells 48 h after the treatment (one experiment in triplicate). BrdU, Bromodeoxyuridine; DMF, dimethyl fumarate; HMC3, human microglial clone 3; LPS, lipopolysaccharide

The percentage of BrdU‐positive HMC3 cells (Figure 4a–c) did not significantly change in non‐activated (29.99 ± 1.29) and CT samples of LPS‐activated and LPS + DMF cells (*p* = 0.30 for both comparisons).

After 48 h of gas treatment, no significant difference in the percentage of BrdU‐positive HMC3 cells was found among CT and gas‐treated samples in both LPS‐activated and LPS + DMF cells (*p* = 0.06 for both).

### 3.3 Wound healing assay

**FIGURE 5:**
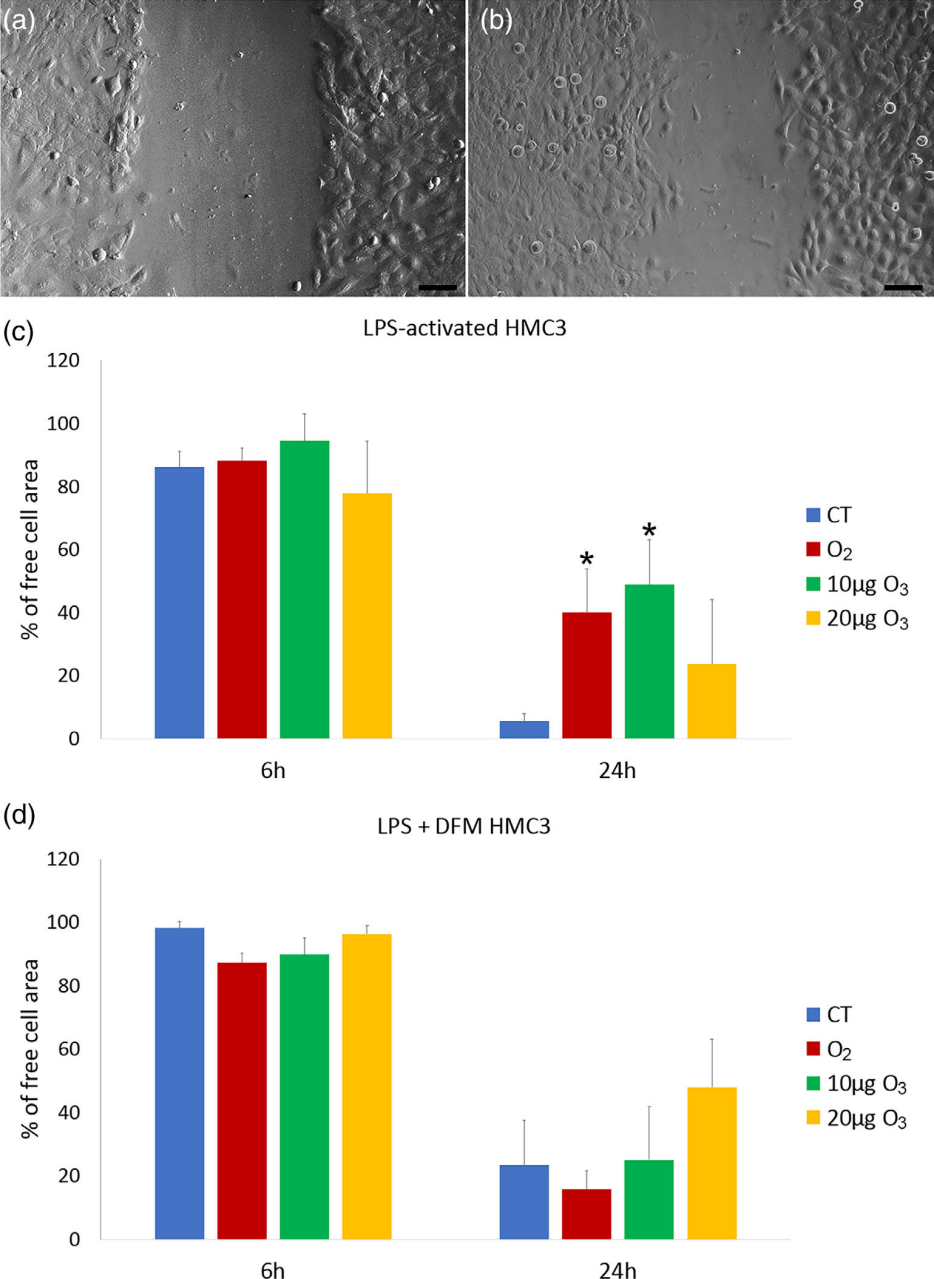
Representative images at inverted microscopy of HMC3 cells at 6 h (a) and 24 h (b) of the wound healing assay. Bars, 100 μm. Means ± SD of percentages of cell‐free areas of CT, O_2_‐ and O_3_‐treated LPS‐activated (c) and LPS + DFM (d) HMC3 cells at 6 h and 24 h of the wound healing assay. Asterisks (*) indicate the statistically significant difference from the corresponding CT samples (*p* < 0.05). DMF, dimethyl fumarate; HMC3, human microglial clone 3; LPS, lipopolysaccharide

The effect of gas exposure on the migration capability of LPS‐activated and LPS + DMF cells was evaluated by the wound healing assay (representative images in Figure 5).

In comparison to non‐activated cells (90.52 ± 3.81), LPS‐activated CT cells showed no significant difference in migration rate at 6 h (*p* = 0.06); instead, LPS + DFM CT cells showed lower migration rates in comparison to both non‐activated and LPS‐activated CT cells (*p* = 0.03). At 24 h, LPS‐activated CT cells showed a significant increase in migration rate in comparison to the non‐activated ones (45.67 ± 10.45) (*p* = 0.03), whereas no statistical difference was found versus LPS + DMF cells (*p* = 0.06).

In LPS‐activated cells (Figure 5c), no statistically significant difference was found in migration rate among CT and gas‐treated samples at 6 h post‐treatment (*p* = 0.79), while at 24 h the migration rate of CT sample was significantly higher in comparison with O_2_‐ and 10 μg O_3_‐treated samples (*p* = 0.03).

In LPS + DFM cells (Figure 5d), no statistical difference was found among CT and gas‐treated samples at both 6 and 24 h.

After 48 h, the wound was completely healed in all samples (not shown).

### 3.4 Cell morphology

The cell shape, surface protrusions, and organelle organization of HMC3 cells were observed by bright field microscopy, SEM, and TEM, respectively.

**FIGURE 6:**
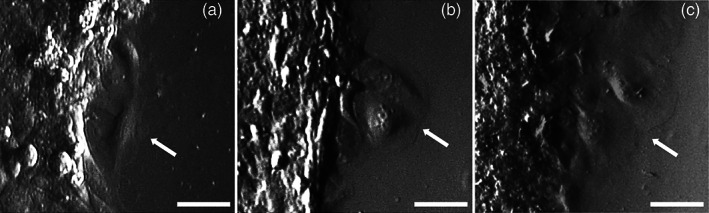
Representative inverted microscope images of spindle‐like non‐activated HMC3 cells (a) and round‐shaped cells in LPS‐activated (b) and LPS + DMF (c) HMC3 cells (arrows). Bars, 50 μm. DMF, dimethyl fumarate; HMC3, human microglial clone 3; LPS, lipopolysaccharide

At inverted microscopy, HMC3 cells showed a flattened and spindle‐like shape in all the conditions investigated, without evident modifications following gas treatments (Figure 6a). However, in LPS‐activated and LPS + DMF samples large flat round‐shaped cells were observed (Figure 6b,c).

**FIGURE 7:**
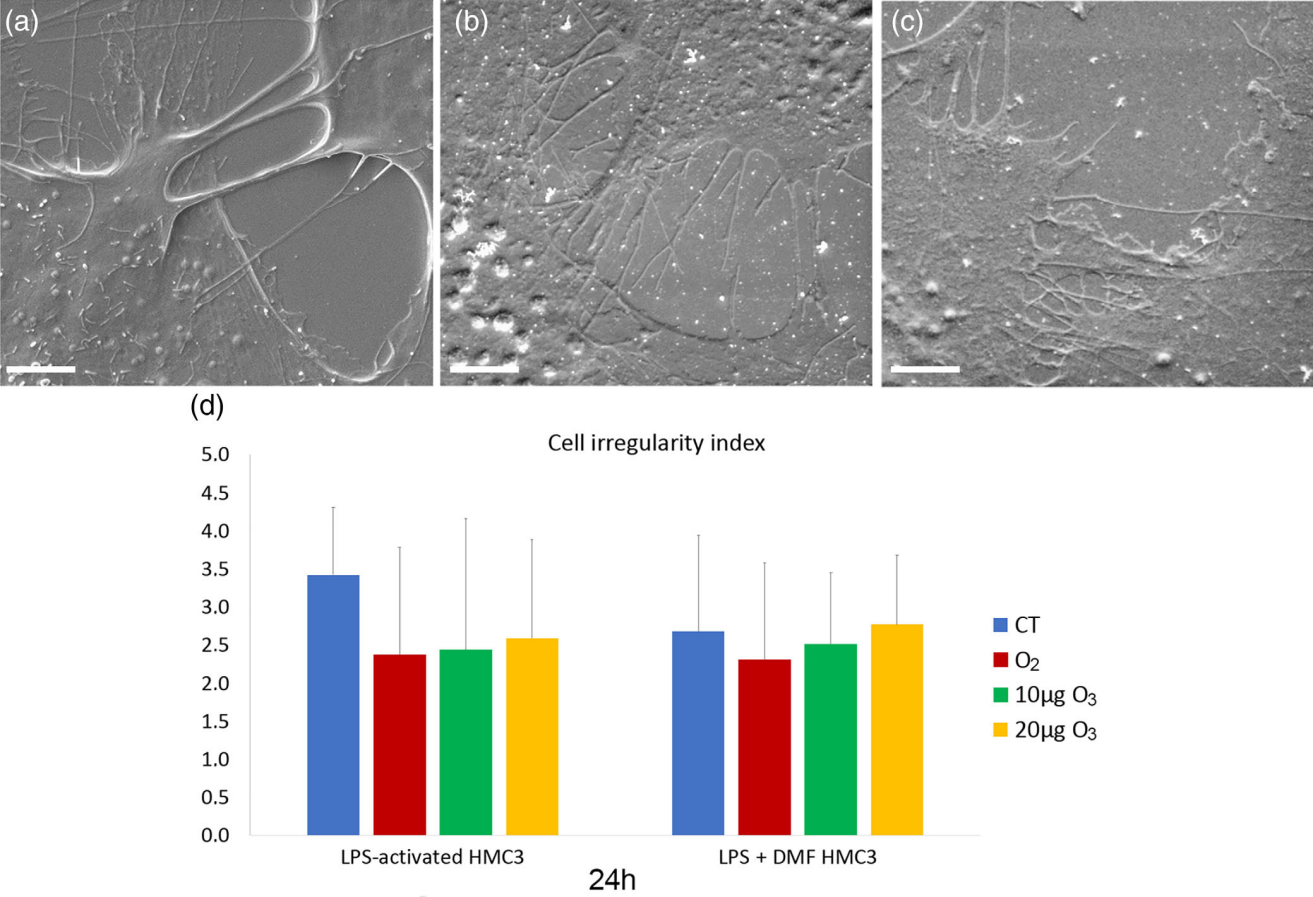
Examples of SEM micrographs of non‐activated cells (a), LPS‐activated (b) and LPS + DMF CT cells. Bars, 5 μm. Means ± SD of the cell irregularity index at 24 h after the treatment (d). DMF, dimethyl fumarate; LPS, lipopolysaccharide; SEM, scanning electron microroscopy

SEM observation showed many thin surface protrusions in all cell samples (Figure 7a–c), independently on activation, and gas treatment. The quantitative evaluation of the surface irregularity did not show significant differences between non‐activated cells (2.54 ± 0.88) and CT samples of LPS‐activated and LPS + DFM cells (*p* = 0.07). Moreover, gas treatment did not induce significant change in cell surface irregularity in LPS‐activated (*p* = 0.18) and LPS + DFM cells (*p* = 0.73) (Figure 7d).

**FIGURE 8:**
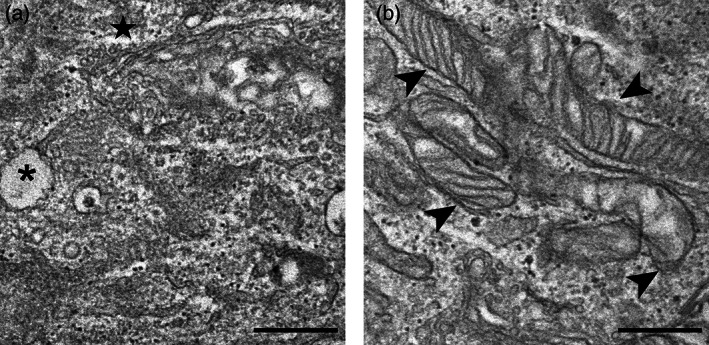
TEM micrographs of non‐activated cells (a–b). Note the cytoplasm rich in smooth endoplasmic reticulum and elongated mitochondria (arrowheads) with lamellar cristae. Asterisk (*) indicates a lipid droplet; star indicates Golgi complex. Bars, 500 nm. TEM, transmission electron microroscopy

**FIGURE 9:**
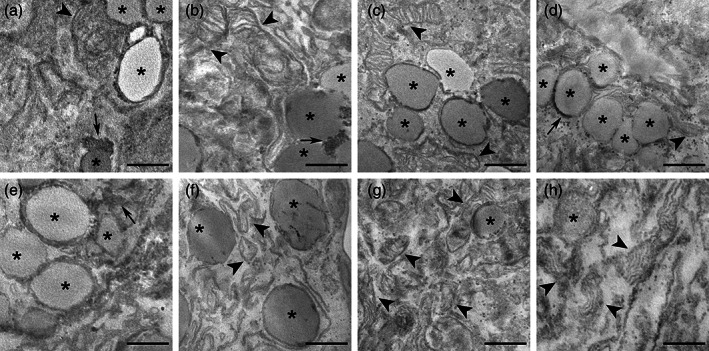
TEM micrographs of LPS‐activated (a–d) and LPS + DMF (e–h) HMC3 cells. CT cells (a, e); O_2_‐treated cells (b, f), 10 μg O_3_‐treated cells (c, g); 20 μg O_3_‐treated cells (d, h). Arrowheads indicate mitochondria. Note the accumulation of lipid droplets (asterisks) and glycogen (arrows) in LPS‐activated and LPS + DMF cells. Bars, 500 nm. DMF, dimethyl fumarate; HMC3, human microglial clone 3; LPS, lipopolysaccharide; TEM, transmission electron microroscopy

TEM provided information on the fine structural organization of HMC3 cells (Figures 8 and 9). Non‐activated cells showed one nucleus; the cytoplasm was characterized by well‐preserved Golgi complex, abundant smooth endoplasmic reticulum, numerous free ribosomes, rare small lipid droplets, and glycogen granules (Figure 8a). Elongated mitochondria with developed lamellar cristae were distributed in the cytoplasm (Figure 8b).

Compared with the non‐activated sample (Figure 8), in the CT samples of LPS‐activated (Figure 9a) and LPS + DMF (Figure 9e) cells, the cytoplasm density decreased and many glycogen granule clusters were often associated with the numerous lipid droplets. In LPS‐activated cells (Figure 9a–d) and in LPS + DMF cells (Figure 9e–h) gas‐treated samples were similar with their respective CT.

### 3.5 Nrf2 distribution

**FIGURE 10:**
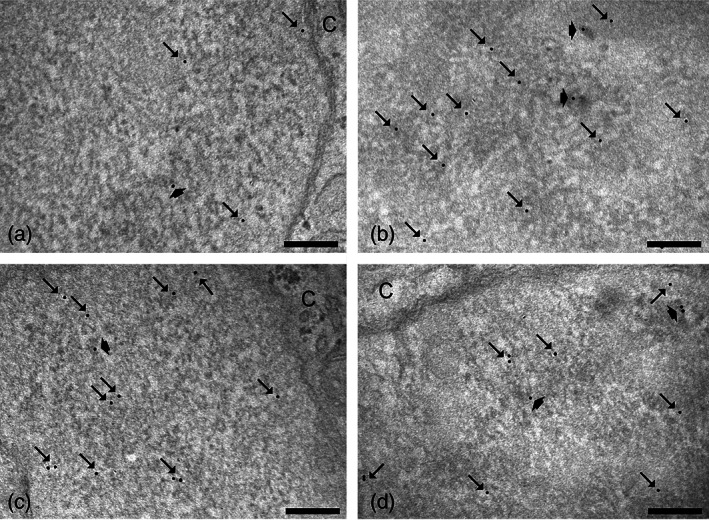
Representative TEM micrographs of HMC3 cell nuclear details after immunolabeling of Nrf2: CT (a) and 20 μg O_3_ (b) of LPS‐activated cells; CT (c) and 20 μg O_3_ (d) of LPS + DMF cells. Immunogold labeling (arrows) occurs on euchromatic regions, especially on perichromatin fibrils (thick arrows). C, cytoplasm. Bars, 200 nm. DMF, dimethyl fumarate; HMC3, human microglial clone 3; LPS, lipopolysaccharide; TEM, transmission electron microroscopy

To assess whether the O_3_ treatment might affect the nuclear distribution of the transcription factor Nrf2, we investigated the ultrastructural immunolabeling of HMC3 cell nuclei. In all samples, Nrf2 was distributed in the euchromatin space (Figure 10), especially on perichromatin fibrils where RNA transcription takes place (Niedojadlo et al., 2011).

Quantitative evaluation of the Nrf2 density revealed that LPS + DMF CT showed similar values to non‐activated cells (0.86 ± 0.32) (*p* = 0.33), whereas in LPS‐activated CT was significantly lower (*p* = 0.001). LPS‐activated CT also revealed lower values in comparison to LPS + DMF CT (*p* = 0.02).

**FIGURE 11:**
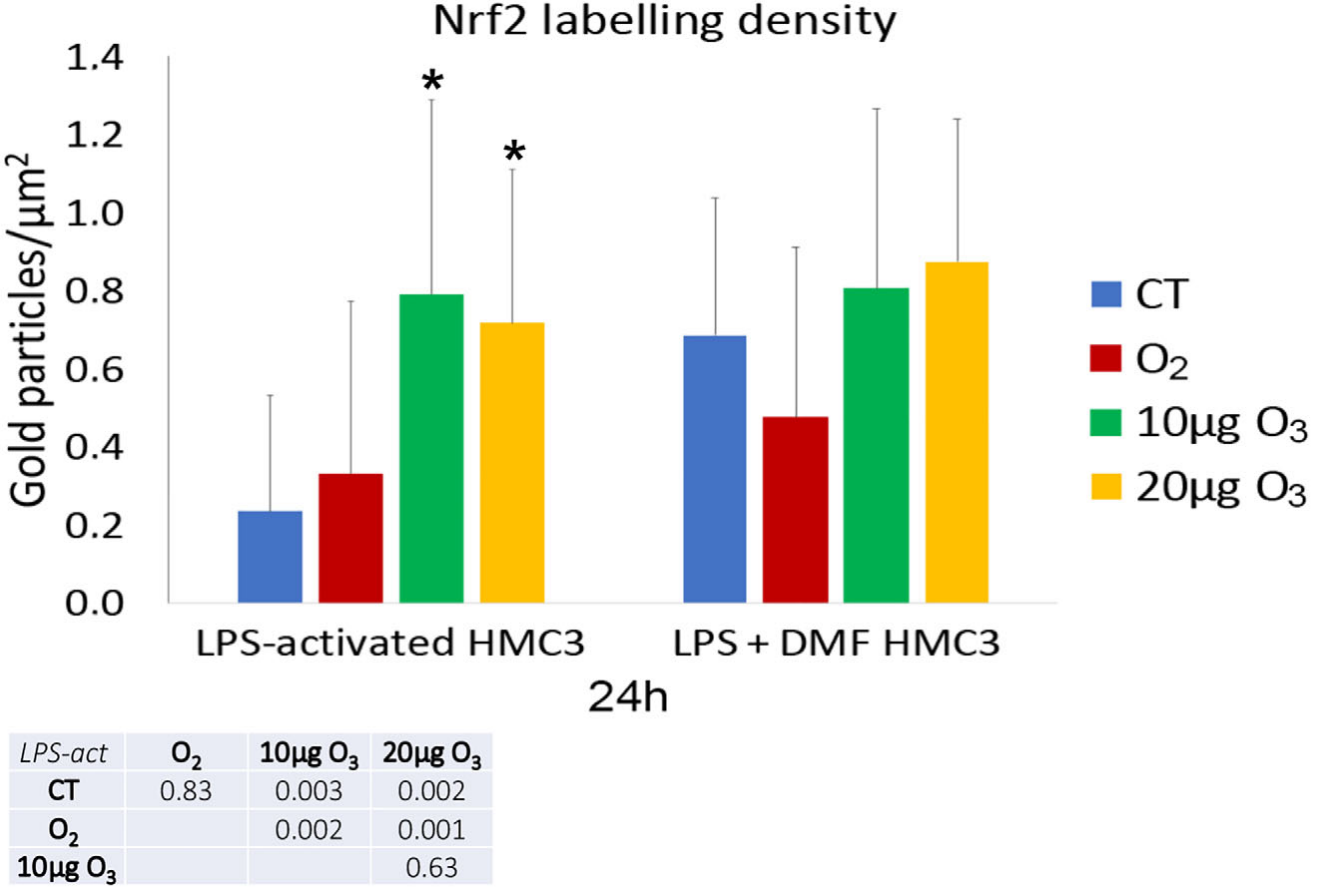
Mean value ± SD of anti‐Nrf2 labeling 24 h after treatment. The table reports the *p* values for all the comparisons made. Asterisks (*) indicate the statistically significant difference from the corresponding CT samples (*p* < 0.05). DMF, dimethyl fumarate; HMC3, human microglial clone 3; LPS, lipopolysaccharide; Nrf2, nuclear factor erythroid 2‐related factor 2

As shown in Figure 11, in LPS‐activated cells, both 10 and 20 μg O_3_‐treated cells showed an increase of the nucleoplasmic anti‐Nrf2 labeling density in comparison with CT and O_2_‐treated cells. In LPS + DMF cells, no statistically significant difference was found among the samples (*p* = 0.45).

### 3.6 Heme oxygenase 1

**FIGURE 12:**
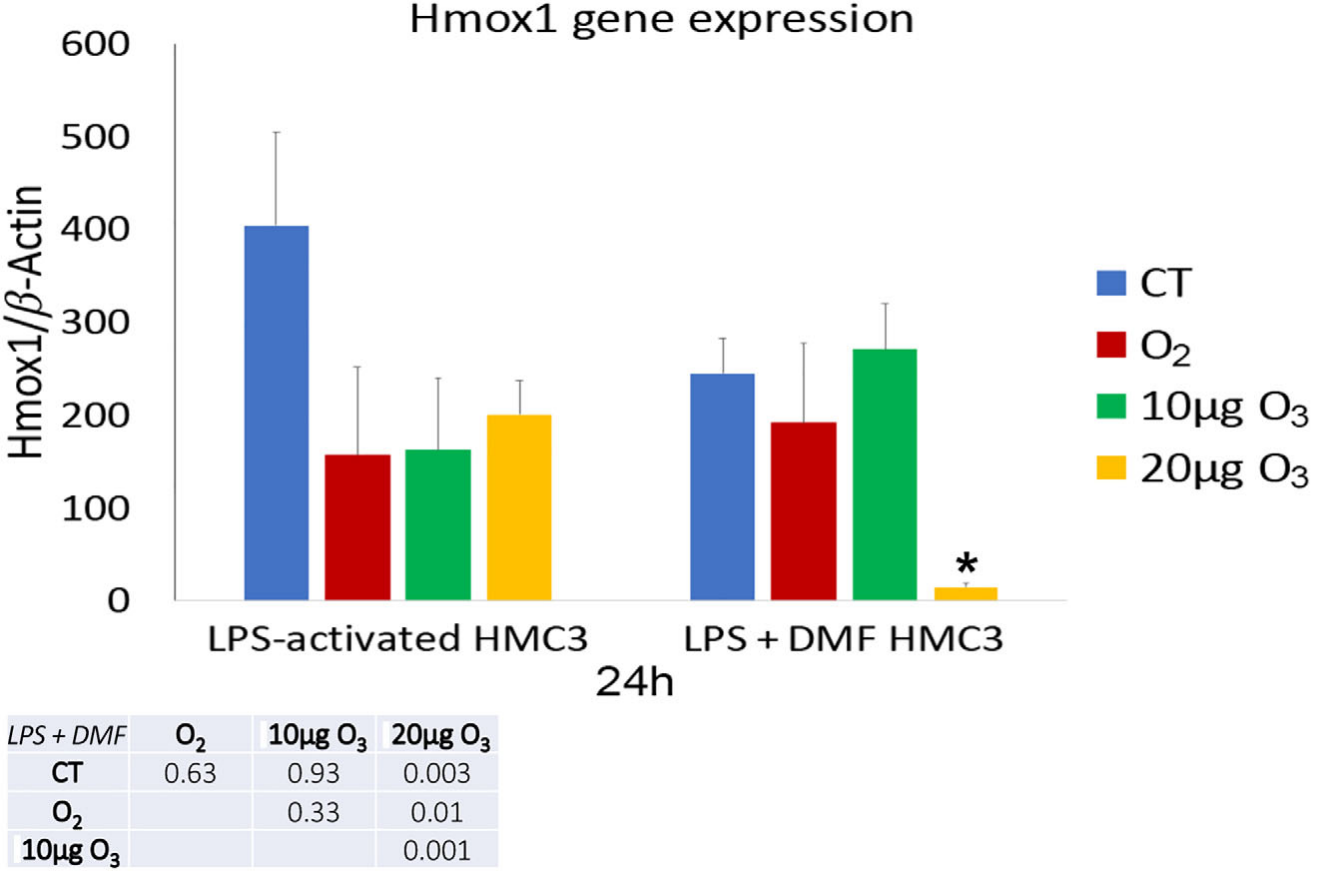
Mean value ± SD of Hmox1 expression at 24 h after treatment. Asterisk (*) indicates the statistically significant difference from the corresponding CT sample (*p* < 0.05). DMF, dimethyl fumarate; HMC3, human microglial clone 3; Hmox1, Heme oxygenase 1; LPS, lipopolysaccharide

Heme oxygenase 1 (Hmox1) gene expression (Figure 12), assessed by Real‐time quantitative polymerase chain reaction (RT‐PCR), resulted significantly higher in both LPS‐activated CT and LPS + DMF CT cells when compared with non‐activated samples (48.50 ± 24.48) (*p* = 0.027).

In both LPS‐activated and LPS + DMF cells no significant difference was found in Hmox1 gene expression among CT and gas‐treated samples, apart from a significant lowering in LPS + DMF cells treated with 20 μg O_3_ (Figure 12).

### 3.7 IL‐6, TNF‐*α*, and IL‐13 secretion

**FIGURE 13:**
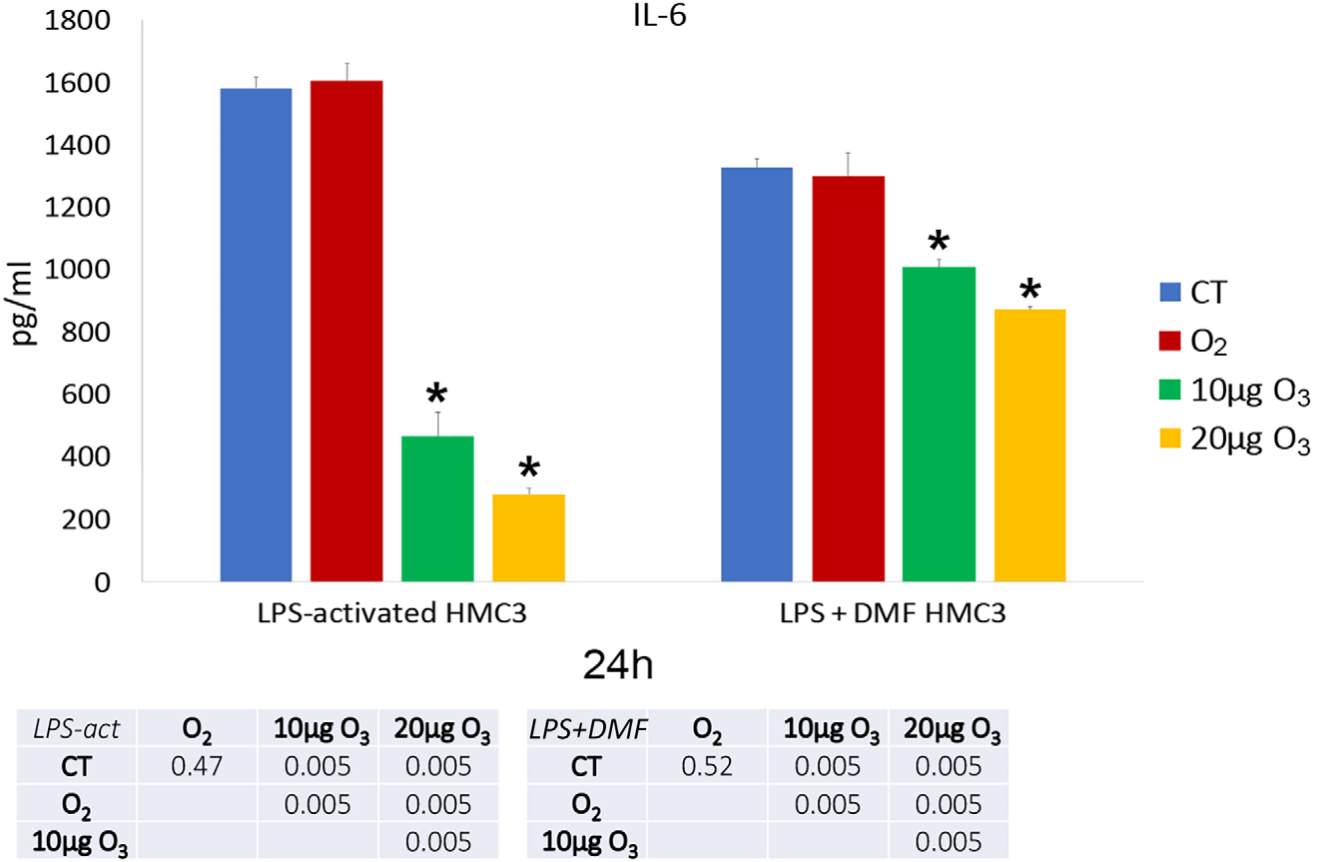
IL‐6 amounts (mean values ± SD) detected in the medium of LPS‐activated and LPS + DMF HMC3 cell samples 24 h after gas treatment (two experiments in duplicate). The table reports the *p* values for all the comparisons made. Asterisks (*) indicate the statistically significant difference from the corresponding CT samples (*p* < 0.05). DMF, dimethyl fumarate; HMC3, human microglial clone 3; IL‐6, Interleukin‐6; LPS, lipopolysaccharide

**FIGURE 14:**
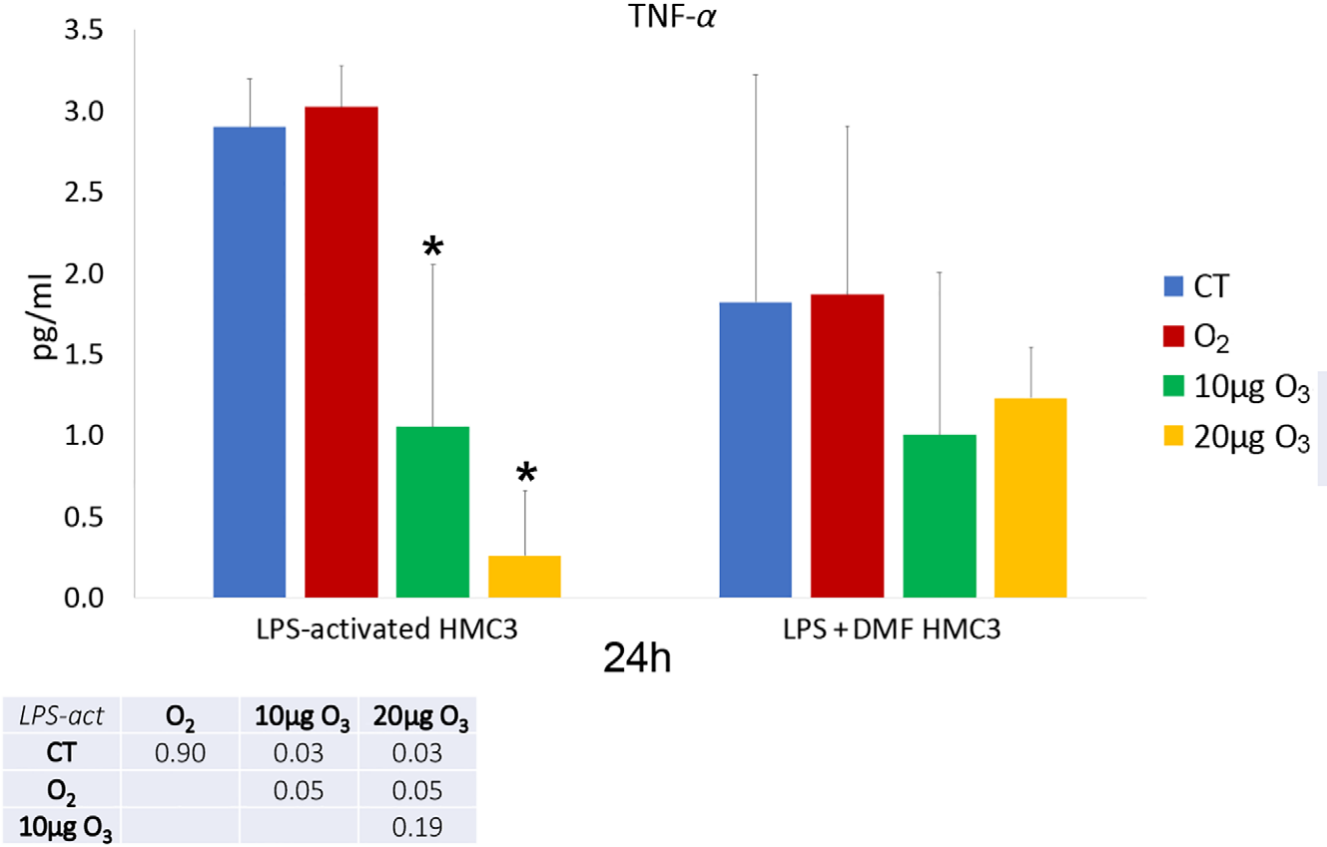
TNF‐*α* amounts (mean values ± SD) detected in the medium of LPS‐activated and LPS + DMF HMC3 cell samples 24 h after gas treatment (two experiments in duplicate). The table reports the *p* values for all the comparisons made. Asterisks (*) indicate the statistically significant difference from the corresponding CT samples (*p* < 0.05). DMF, dimethyl fumarate; HMC3, human microglial clone 3; LPS, lipopolysaccharide; TNF‐*α*, tumor necrosis factor‐*α*

**FIGURE 15:**
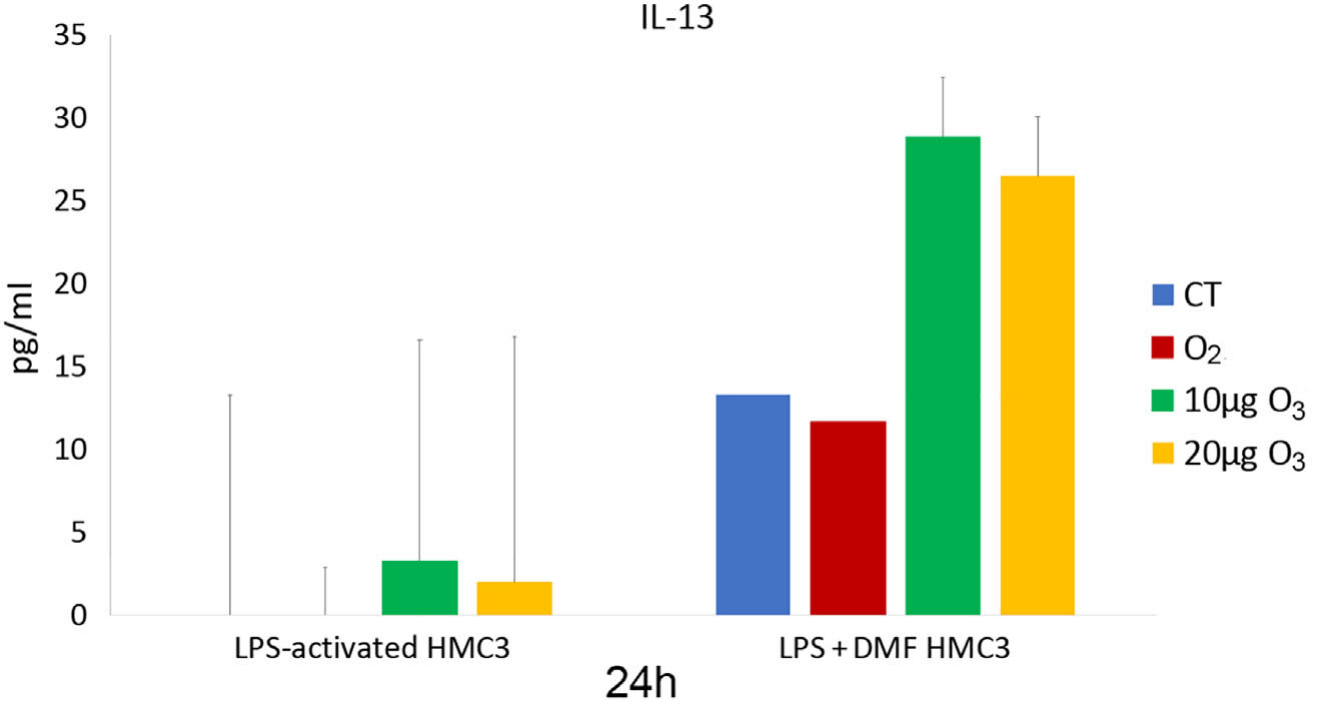
IL‐13 amounts (mean values ± SD) detected in the medium of LPS‐activated and LPS + DMF HMC3 cell samples 24 h after gas treatment (two experiments in duplicate). DMF, dimethyl fumarate; HMC3, human microglial clone 3; IL‐13, Interleukin‐13; LPS, lipopolysaccharide

The amount of IL‐6 (Figure 13) and TNF‐*α* (Figure 14) as pro‐inflammatory cytokines, and IL‐13 (Figure 15) as an anti‐inflammatory cytokine was evaluated in the culture medium of LPS‐activated and LPS + DMF HMC3 cells, in order to assess their secretory activity.

As for IL‐6, LPS‐activated CT cells showed significantly higher values in comparison to non‐activated cells (695.45 ± 34.98) (*p* = 0.005). LPS + DMF CT cells showed values significantly higher than non‐activated cells (*p* = 0.004) but significantly lower than CT of LPS‐activated cells (*p* = 0.004). As shown in Figure 3, in LPS‐activated conditions and in LPS + DMF samples both 10 and 20 μg O_3_‐treated cells showed significantly lower values than CT and O_2_‐treated cells.

As for TNF‐*α*, CT samples of LPS‐activated and LPS + DMF cells showed significantly higher values in comparison to non‐activated cells (0.98 ± 0.29) (*p* = 0.02 and *p* = 0.03, respectively); moreover, CT sample of LPS + DMF cells showed significantly lower values than CT of LPS‐activated cells (*p* = 0.04). In LPS‐activated conditions (Figure 14), both 10 and 20 μg O_3_‐treated samples showed significantly lower values than CT and O_2_‐treated cells, while no significant difference was found among cell samples in LPS + DMF conditions (*p* = 0.08).

As for IL‐13 (Figure 15), CT samples of LPS‐activated cells showed values below the detection limit, while CT samples of LPS + DMF cells showed values significantly lower that non‐activated cells (31.08 ± 13.25) (*p* = 0.03). No significant difference was found among cell samples in LPS‐activated and LPS + DMF conditions (*p* = 0.66 and *p* = 0.06, respectively).

## 4 DISCUSSION

O_2_‐O_3_ therapy proved to be beneficial in some neurodegenerative diseases characterized by high oxidative stress and inflammation (Delgado‐Roche et al., 2017; Izadi et al., 2020; Lin et al., 2019; Lintas et al., 2013; Tahmasebi et al., 2021). These promising data require a deep knowledge of the O_3_ action mechanisms on microglial cells, which play multiple roles for the maintenance of homeostasis in the CNS and are primarily involved in both neuroinflammatory and neuroprotective processes in many neurodegenerative diseases (Ho, 2019; Kwon & Koh, 2020; Leng & Edison, 2021; Voet et al., 2019).

First, our findings demonstrated that the in vitro experimental model used in the present study is reliable. In fact, consistent with previous findings on activated microglial cells, LPS‐activated HMC3 cells showed increased migration rate (Dello Russo et al., 2018), morphological modifications from elongated/branched to larger roundish cells (Baek et al., 2021; Garcia‐Contreras & Thakor, 2021), lipid accumulation in the cytoplasm (Khatchadourian et al., 2012), decreased cytoplasmic and mitochondrial electron density (Anderson et al., 1995), decreased Nrf2 amounts, and increased Hmox1 gene expression and secretion of the pro‐inflammatory cytokines IL‐6 and TNF‐*α* (Pallio et al., 2021). Treatment with DMF – a neuroprotective drug acting through Nrf2 (Scannevin et al., 2012) – proved to mitigate the alterations in LPS‐activated cells, partially restoring some of the structural and functional features of non‐activated HMC3 cells.

Gas treatment did not alter cell viability in both LPS‐activated and LPS + DMF cells (apart from a lower vitality after 72 h from the treatment with 20 μg O_3_ in LPS + DMF cells). These findings are in agreement with reports in the literature demonstrating that these low O_3_ concentrations are safe for many cell types (Cisterna et al., 2021; Costanzo et al., 2018; Costanzo et al., 2015; Scassellati et al., 2017). However, HMC3 cells proved to be especially sensitive to oxidative stress since a concentration of 30 μg of O_3_, which is tolerated by most cell types under similar experimental conditions, were lethal to them.

Similarly, gas exposure did not affect proliferation in both LPS‐activated and LPS + DMF cells, as shown by the evaluation of both the mitotic index and BrdU‐positivity, according to previous reports on other cell types in vitro (Costanzo et al., 2020; Costanzo et al., 2015; Scassellati et al., 2017).

The safety of 10 and 20 μg O_3_ for HMC3 cells was unequivocally testified by the high‐resolution analysis at TEM and SEM. In fact, while LPS treatment induced in microglial cells various morphological modifications in comparison to control (as highlighted above), gas exposure did not affect any feature in both LPS‐activated and LPS + DMF cells.

Cell motility represents a hallmark of microglial cell activation (Baek et al., 2021; Garcia‐Contreras & Thakor, 2021; Zhang et al., 2016). Accordingly, after LPS treatment, some HMC3 cells showed a large roundish cell body, characteristic of activated microglia able to ameboid movement, while the wound healing test demonstrated that LPS‐activated cells had a higher migration rate in comparison to non‐activated cells. As for the effect of gas exposure, in LPS‐activated cells both O_2_ and 10 μg O_3_ decreased significantly cell motility while cell surface protrusions, involved in cell migration (Fraley et al., 2010), showed a slight although not significant tendency to reduce. This pointed out the reduction of one typical feature of the inflammation phenotype. The reasons for such an effect remain unclear but it could be related to the influence of oxidant‐antioxidant balance on cytoskeleton dynamics (Muliyil & Narasimha, 2014); studies on this topic are currently in progress in our laboratories.

Similarly to other cell types treated with low O_3_ concentrations (Cappellozza et al., 2021; Galiè et al., 2018), in LPS‐activated cells Nrf2 translocated to the nucleus following the mild oxidative stress due to 10 and 20 μg O_3_ exposure. However, in HMC3 cells this translocation did not lead to an increase in the transcription of Hmox1 gene, the gene marker of the antioxidant response following O_3_ treatment (Scassellati et al., 2017), thus revealing a lower responsiveness of these microglial cells to the antioxidant action of O_3_ in comparison to other cell types submitted to the same gas treatment (Cappellozza et al., 2021; Cisterna et al., 2021; Scassellati et al., 2017). In LPS + DMF cells no difference in the amount of nuclear Nrf2 was induced by gas exposure. Since the quantity of nuclear Nrf2 found in LPS + DMF CT cells was higher than in LPS‐activated CT cells, it can be hypothesized that O_3_ has no additional effect to the nuclear translocation induced by the treatment with DMF (Scannevin et al., 2012). Consistently, no increase in Hmox1 expression was induced by the gas treatment in LPS + DMF cells. It is worth noting that Nrf2 has multiple direct and indirect effects on manifold pathways concerning not only the antioxidant response but also, for example, mitochondrial bioenergetics, unfolded protein response, proteasome activity, intermediary metabolism regulation, stem cell proliferation, and differentiation (Tonelli et al., 2018). This opens broad prospects for studies on the Nrf2‐driven effects of O_3_.

Cytokine secretion is another typical hallmark of microglia activation in vivo and in vitro (Prinz et al., 2019; Wolf et al., 2017; Woodburn et al., 2021). LPS is an inflammatory agent known to increase IL‐6 and TNF‐*α* secretion in HMC3 (Dello Russo et al., 2018; Garcia‐Contreras & Thakor, 2021; Lu et al., 2021). IL‐6 and TNF‐*α* are pro‐inflammatory cytokines: under physiological conditions, they are scarcely present, but in many CNS pathologies or injury they are produced in high amount by various immune cells including microglia, playing multiple roles in neurorepair (Rothaug et al., 2016; Schroeter & Jander, 2005; Welser‐Alves & Milner, 2013). However, in the presence of chronic neuroinflammation, their excessive secretion becomes detrimental and may promote the development of neurodegenerative diseases (Kaur et al., 2019; Lyman et al., 2013; Shabab et al., 2017; Smith et al., 2012). On the other hand, IL‐13 is an anti‐inflammatory cytokine mostly secreted by lymphocytes (Minty et al., 1993) and recently found to also be produced, although in small amount, by HMC3 (Caruso et al., 2021; Pallio et al., 2021).

In LPS‐activated HMC3 cells, exposure to low O_3_ concentrations induced a marked decrease in IL‐6 secretion. Similar results were found for TNF‐*α*. On the other hand, pure O_2_ seems to be ineffective in modulating both cytokines, demonstrating the direct involvement of O_3_ in decreasing the secretion of pro‐inflammatory cytokines in activated HMC3 cells. Such modulatory action of low O_3_ concentration on cytokine secretion has been already documented both in vivo and in vitro (Cappellozza et al., 2021; Cisterna et al., 2021; Delgado‐Roche et al., 2017; Güçlü et al., 2016; Tahmasebi et al., 2021; Tartari et al., 2020; Zeng et al., 2020).

Interestingly, the effect of low O_3_ concentrations on pro‐inflammatory cytokines is similar to that induced by the anti‐inflammatory drug DMF. This could be because both O_3_ and DMF act through Nrf2 (Scannevin et al., 2012), likely activating similar cytoprotective pathways. It is worth noting that, as reported in a previous work (Pallio et al., 2021), activated HMC3 reduced IL‐6 and TNF‐*α* secretion after treatment with Metaxalone, a drug that also induces Nrf2 increase. Low O_3_ concentrations proved to significantly decrease IL‐6 secretion even in LPS + DMF cells, thus suggesting an adjuvant role.

The secretion of IL‐13 from non‐activated HMC3 cells was quite scarce. LPS activation induced a drastic lowering of IL‐13 secretion (that became undetectable in the medium), and the slight IL‐13 increase after exposure to low O_3_ concentrations was statistically insignificant. In LPS + DMF CT cells, the exposure to DMF restored the secretion of IL‐13, similarly to what found by Pallio et al., 2021 after treating HMC3 cells with Metaxalone, but again the O_3_ treatment did not change IL‐13 secretion.

In conclusion, the combined application of refined microscopical and biomolecular techniques to activated microglial HMC3 cells confirmed that low‐dose O_3_ does not induce structural alterations while being able to decrease cell migration and the secretion of pro‐inflammatory cytokines. Interestingly, these responses to O_3_ treatment do not involve the upregulation of antioxidant genes such as Hmox1, but anyway imply the activation of Nfr2, which is known to act through many pathways besides the antioxidant ones.

The results we obtained in this simplified system in vitro allowed elucidating a basic cell mechanism, suggesting that the modulation of microglia activity may contribute to the beneficial effects of the O_2_‐O_3_ therapy in patients affected by neurodegenerative disorders characterized by chronic inflammation.

## CONFLICT OF INTEREST

The authors declare no conflicts of interest.

## Data Availability

The data that support the findings of this study are available from the corresponding author upon reasonable request.
